# Exploring novel blood-based DNA methylation biomarkers for alzheimer’s disease via targeted sequencing of highly variable CpG sites

**DOI:** 10.1186/s13104-025-07417-7

**Published:** 2025-08-12

**Authors:** Hideki Ohmomo, Shohei Komaki, Shiori Minabe, Yoichi Sutoh, Yayoi Otsuka-Yamasaki, Makoto Sasaki, Atsushi Shimizu

**Affiliations:** 1 Division of Biomedical Information Analysis, Institute for Biomedical Sciences, Iwate Medical University, 1-1-1 Idaidori, Yahaba, 028-3694 Shiwa, Iwate Japan; 2 Disaster Reconstruction Center, Iwate Tohoku Medical Megabank Organization, Iwate Medical University, 1-1-1 Idaidori, Yahaba, 028-3694 Shiwa, Iwate Japan; 3 Division of Ultrahigh Field MRI, Institute for Biomedical Sciences, Iwate Medical University, 1-1-1 Idaidori, Yahaba, 028-3694 Shiwa, Iwate Japan

**Keywords:** Alzheimer’s disease, DNA methylation biomarkers, Epigenome-wide association study, Targeted-bisulphite sequencing, *APOE* genotyping

## Abstract

**Objective:**

Dementia, particularly Alzheimer’s disease (AD), continues to be a major public health concern due to population aging, yet minimally invasive biomarkers for early diagnosis have not been established. DNA methylation (DNAm) has recently attracted considerable attention as a promising biomarker. This study aimed to identify blood-based DNAm biomarkers for early detection of AD.

**Results:**

We analysed blood-derived DNA from 48 patients with AD (from Biobank Japan) and 48 age- and sex-matched controls (from the Tohoku Medical Megabank Biobank) using Apolipoprotein ε type 4 (*APOE*)-associated genotype analysis and targeted-bisulfite sequencing. High-risk *APOE* genotypes were more frequent in AD patients (23/48, [47.9%]) than in controls (6/48, [12.5%]). A typical case-control and *APOE* genotype-stratified epigenome-wide association study (EWAS) did not identify any genome-wide significant CpG sites. Although the primary findings were negative, some top CpG sites appeared in both analyses, including loci on the Cell Adhesion Molecule 1 (*CADM1*), Tubulin alpha 1b (*TUBA1B*), and Exocyst complex component 2 (*EXOC2*) genes, which have previously been linked to AD-related pathways. The relatively early clinical stage and uncertainty of disease onset might have limited detection sensitivity. Longitudinal studies with refined staging and multi-omics integration might clarify the biomarker potential of blood DNAm in AD.

**Supplementary Information:**

The online version contains supplementary material available at 10.1186/s13104-025-07417-7.

## Introduction

Alzheimer’s disease (AD) is a type of dementia that primarily affects older adults, causing significant brain tissue damage and the death of nerve cells. This leads to memory loss and difficulties with activities such as reading, writing, speaking, and recognizing close relatives. The prevalence of AD has become a critical societal issue as the global population ages. Effective therapeutic options remain elusive despite considerable efforts to develop disease-modifying drugs and vaccines targeting amyloid-β [1–5] the primary pathological hallmark of AD. Current treatment and prevention strategies primarily focus on mild cognitive impairment (MCI), the preclinical stage of AD, but an MCI diagnosis requires cognitive function tests, positron emission tomography, and magnetic resonance imaging. The US Food and Drug Administration has approved cerebrospinal fluid AD biomarker tests and these are included in the National Institute for Health and Clinical Excellence dementia guidelines [6]. However, these tests involve invasive lumbar puncture. Consequently, simpler and less invasive early diagnostic methods, such as blood tests are urgently needed. Specific blood cell subtypes such as platelets, may contribute to AD-related pathophysiology, such as amyloid processing and inflammation, and serve as accessible sources of biomarkers [7]. 

Genome-wide association studies (GWAS) have identified several AD susceptibility polymorphisms, with Apolipoprotein ε type 4 (*APOE4*) being the most prominent example [8]. Other GWAS and meta-analyses have revealed common variants in multiple genetic loci, including Myc box-dependent-interacting protein 1 (*BIN1*; also known as Bridging Integrator-1 and Amphiphysin-2), Clusterin (*CLU*), Complement Receptor 1 (*CR1*), and Phosphatidylinositol binding clathrin assembly protein (*PICALM*) [9–15]. Japanese population studies have found AD-associated single nucleotide polymorphisms (SNPs) such as Sortilin-related receptor 1 (*SORL1*) [16] and Contactin-associated protein-like 2 (*CLTNAP2*) [17]. However, AD onset prediction based solely on these genetic factors remains challenging and emphasises the need for additional biomarkers that integrate both genetic and environmental factors. 

Lifestyles and environmental exposure can influence DNA methylation (DNAm) profiles and the consequent expression of genes that are associated with the onset of various diseases; hence, they emerged as promising biomarkers [18–21]. Peripheral blood DNAm has the potential for detect preclinical states and the early onset of cancers and mental disorders [22–24]. Although both *APOE* and *TOMM40* [25] are hypomethylated in patients with AD, reliable DNAm biomarkers of early AD have not been developed [26]. One potential limitation is that most reported DNAm biomarkers are based on microarray technologies that cover only ~ 3.5% of CpG sites in the entire genome, suggesting that other biomarkers await exploration.

We previously identified blood-derived DNAm biomarkers of early clear-cell renal cell carcinoma [27] and valvular heart disease [28] that are difficult to diagnose. Building on these results, we aimed to identify novel blood-derived DNAm biomarkers for AD using sequencing-based analyses in the present study.

## Materials and methods

### Ethics

This study complied with the Japanese Ethical Guidelines for Medical and Health Research Involving Human Subjects. Additionally, it was approved by the BioBank Japan (BBJ) Sample and Data Utilization Review Committee (Receipt No. P0214 and P0325) and the Ethics Committee of Iwate Medical University (HG2020-023). We explained the research to the AD patients themselves and obtained their written informed consent. However, in cases where obtaining consent directly from the patient was difficult, proxy consent was obtained from one of the following individuals: the patient’s spouse, adult children, parents, competent adult siblings, adult grandchildren, grandparents, or legal guardians. Written informed consent was also obtained from all healthy controls.

### Study participants

**Fig. 1:**
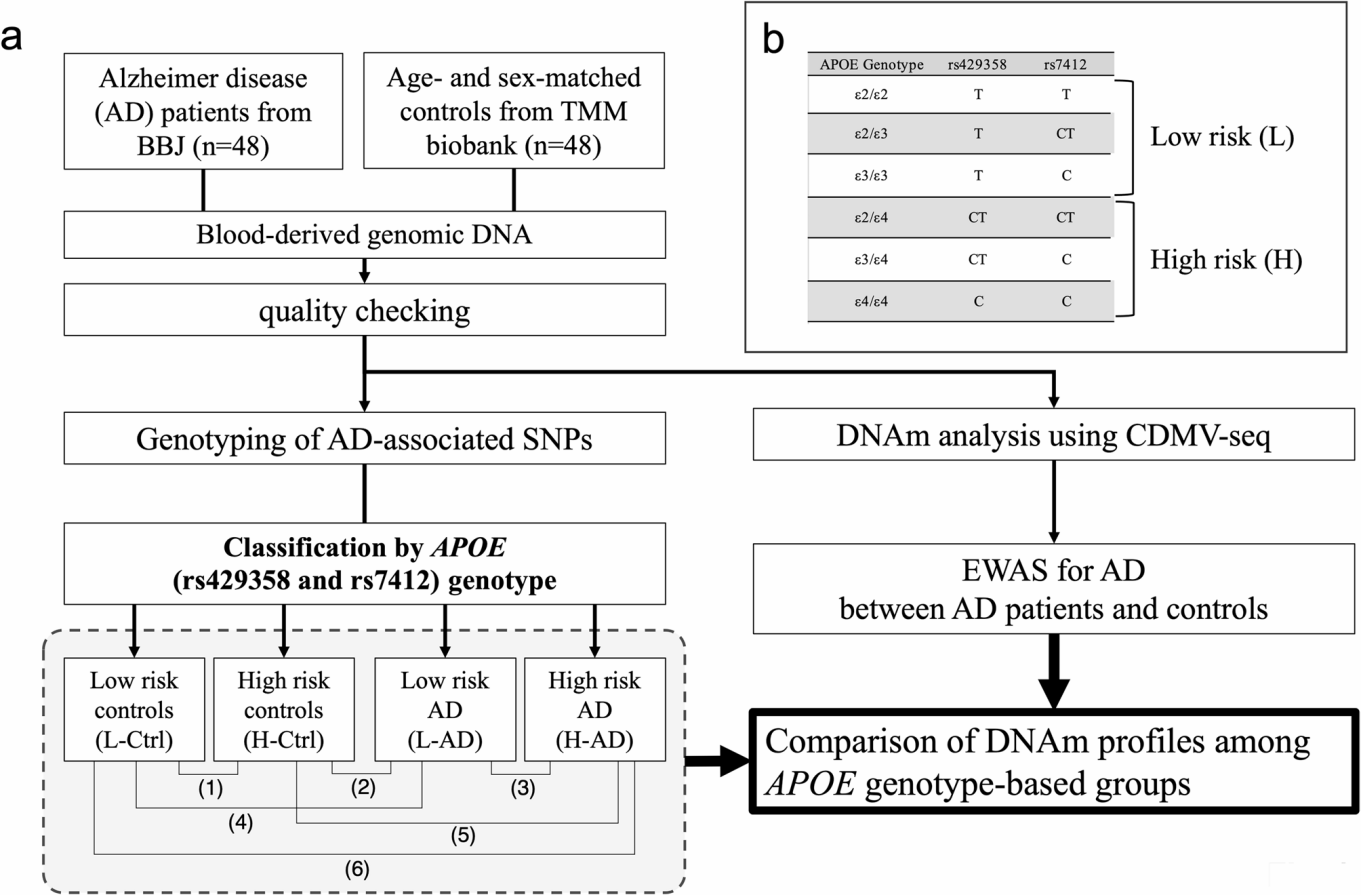
The workflow of this study (**A**). Comparisons of L-Ctrl and H-Ctrl (1), H-Ctrl and L-AD (2), L-AD and H-AD (3), L-Ctrl and L-AD (4), H-Ctrl and H-AD (5), and L-Ctrl and H-AD (6). The definition of AD risk using *APOE*-genotypes (rs429358 and rs7412) (**B**). AD, Alzheimer’s disease patients; CDMV, common DNA methylation, variation; Ctrl, controls; DNAm, DNA methylation; TMM, Tohoku Medical Megabank

We obtained blood-derived DNA from 48 patients with AD from the BBJ, a biobank supported by AMED. All Japanese patients with AD resided in Japan (exact location unknown) and were enrolled in BBJ between October 2013 and September 2017. We obtained 48 age- and sex-matched healthy controls from the Biobank of Tohoku Medical Megabank (TMM) project (Fig. 1). Japanese control participants resided in Iwate prefecture, Japan and were enrolled in the TMM project between July 2013 and March 2016. Blood samples were collected from patients with AD before treatment. Genomic DNA quality control methods are detailed in Supplementary Methods. We used the *TTestIndPower* module from the *statsmodels* package in Python to analyse the power required to detect differential methylation *post hoc*. Assuming a 15% DNAm difference (Cohen’s d = 0.75), a standard deviation (SD) of 20%, and a genome-wide suggestive threshold (α = 5 × 10⁻⁶), the estimated power required to detect differential methylation in samples from 48 participants in each group was ~ 13.8%.

### Selection of AD-associated SNPs

As of March 2022, a search of the ClinVar database for SNPs potentially associated with AD yielded 697 SNPs. The following exclusion criteria were then used: (1) SNPs with “not provide” in the “Condition” column, (2) SNPs with “not provide” in the “Clinical Significance” column, and (3) SNPs with no data in the “Protein Change” column. After PCR primer design and amplification, 76 SNPs from 14 genes were included for genotyping (Supplementary Table S1). The PCR protocol is described in Supplementary Methods.

### Bisulfite sequencing

Sequencing libraries were prepared using SureSelect XT Human Methyl-Seq Kit (Agilent Technologies, Santa Clara, CA, USA), and the probe set contained 1.5 million CpG sites where DNAm profiles varied between individuals (common DNA methylation variations; CDMV). The CDMV probe set is enriched in the genomic regions with inter-individual DNAm variations based on our previous findings of whole genome bisulfite sequencing in purified monocytes, CD4^+^ lymphocytes, and neutrophils [29, 30]. After sequencing using the HiSeq2500 System (Illumina Inc., San Diego, CA, USA), we calculated DNAm levels and processed data Supplementary Methods. Based on our previous findings [27, 30] we analyzed DNAm data with a read depth of ≥ 6×, considering a balance among data quality, coverage, and sequencing cost.

### Typical case-control EWAS for AD and *APOE* genotype-based EWAS

First, we conducted a typical case-control EWAS comparing DNAm profiles between AD and controls to correct for cell-type composition using logistic regression. Based on previous studies of AD risk by *APOE* genotypes [31, 32] the AD and control groups were classified as low risk (ε2/ε2, ε2/ε3, ε3/ε3) and high risk (ε2/ε4, ε3/ε4, ε4/ε4), respectively (Fig. 1b), and intergroup comparison based on *APOE* genotyping was performed as shown in Fig. 1a (1)-(6). DNAm-level calculations and statistical methods are detailed in Supplementary Methods. For the analyses, CpG sites with a high proportion of missing data (≥ 50%) or no interindividual variation were excluded. Significance thresholds were adjusted for multiple comparisons using the Bonferroni correction.

## Results

### Participant characteristics

The characteristics of the study participants are summarised in Table 1. The patients with AD and controls included 15 men and 33 women. The mean age of men and women was 80.0 ± 4.9 and 81.5 ± 5.4 years for patients with AD, and 80.0 ± 5.1 and 81.6 ± 5.2 years for the controls, respectively. The average time since AD diagnosis was 3.15 ± 3.03 years. The clinical symptoms of the patients with AD included visual and auditory hallucinations (*n* = 2), delusions (*n* = 3), day/night reversal (*n* = 2), verbal abuse (*n* = 4), and violent behaviour (*n* = 2). The Clinical Dementia Rating scale [33, 34] scores were 0 or 0.5, 1, 2, 3, and unknown in two, 10, 12, five, and 19 patients, respectively.

**Table 1:** Characteristics of the participants

			AD patients				Controls	
Enrolment period		Oct. 2013	–	Sep. 2017		Jul. 2013	–	Mar. 2016
Sex (N)								
	Male		15				15	
	Female		33				33	
Age (mean ± SD)								
	Male	80.0	±	4.9		80.0	±	5.1
	Female	81.5	±	5.4		81.6	±	5.2
Visual and auditory hallucinations*(N*)			2				−	
Delusion(*N*)			3				−	
Day-night reversal(*N*)			2				−	
Abusive language(*N*)			4				−	
Violence(*N*)			2				−	
Clinical Dementia Rating scale^*1^(*N*)								
	0		1				−	
	0.5		1				−	
	1		10				−	
	2		12				−	
	3		5				−	
	NA		19				−	
Mean gap (y) since AD Diagnosis (± SD)		3.15	±	3.03			-	
*APOE* genotyping (*N*)								
	ε2/ε2		1				0	
	ε2/ε3		1				3	
	ε3/ε3		23				39	
	ε2/ε4		1				0	
	ε3/ε4		20				6	
	ε4/ε4		2				0	

*1 The Clinical Dementia Rating scale scores the severity of dementia as none (0), questionable (very mild; 0.5) mild (1), moderate (2), or severe (3)

### Genotyping for AD-associated SNPs

*APOE* (rs429358 and rs7412) genotyping revealed that the proportion of high-risk individuals was higher in the AD group than in the controls (23 in 48 patients with AD vs. 6 in 48 controls) (Table 1). The other AD-associated SNP genotyping results are shown in Supplementary Table S2. Significant differences between patients with AD and controls were observed for five SNPs: *TF* (rs1049296), *NOS* (rs1799983), *PSEN1* (rs63751139 and chromosome (Chr.) 14; position (pos.)73683951), and *PRNP* (rs1799990).

### DNAm analysis, case-control EWAS for AD, and *APOE* genotype-based EWAS

**Fig. 2:**
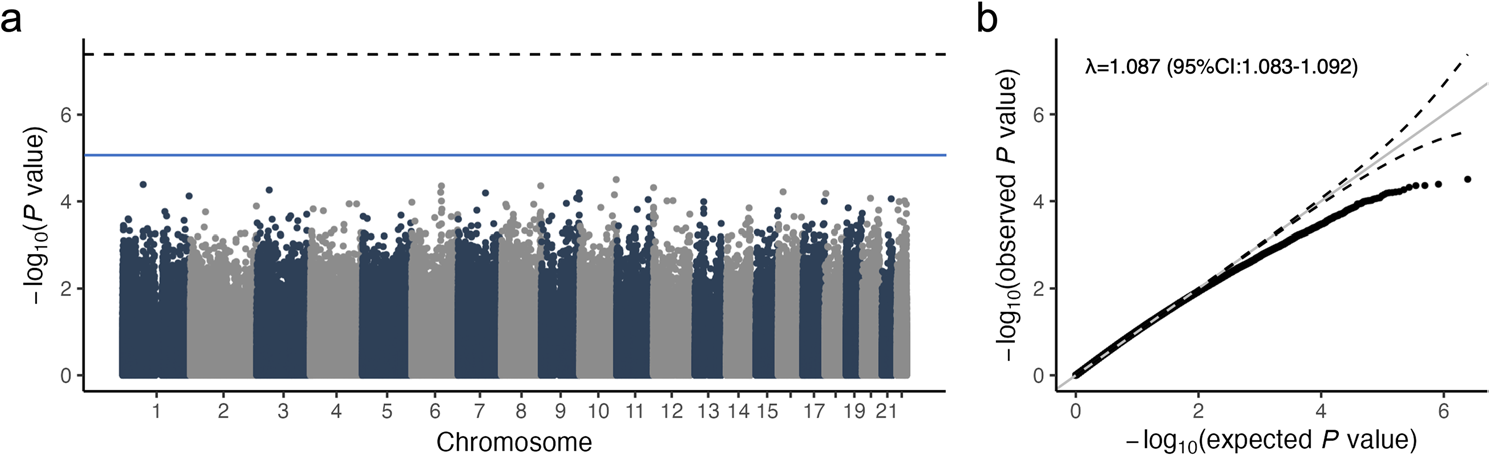
The Manhattan (**A**) and quantile-quantile (QQ) (**B**) plots of typical case-control EWAS for AD. The blue and dashed lines show the suggestive (*p* < 1.00 × 10^− 5^) and Bonferroni lines (*p* < 5.62 × 10^− 8^), respectively. λ, inflation factor; 95%CI, 95% confidence interval

Of the 1.5 million CpG sites on our CDMV probes, approximately 1.24 million (82.7%) had a read depth ≥ 6× (Supplementary Table S3). No bias was observed in the DNAm profiles between patients with AD and controls or among *APOE* genotype groups (Supplementary Figs. S1a and S1b). As the estimated proportions of six blood cell types (monocytes, neutrophils, CD4^+^ T cells, CD8^+^ T cells, NK cells, and B cells) were associated with the first five principal components (PCs), regression analyses included PCs 1–5 as covariates (Supplementary Fig. S1c). The case-control EWAS for AD revealed no significant CpG sites (Fig. 2a; adjusted *p* < 5.62 × 10^− 8^, Bonferroni correction). The comparison of DNAm profile data with the theoretical distribution showed weak inflation (λ = 1.087, 95% CI: 1.083–1.092) (Fig. 2b). Although none of the top 30 CpG sites were previously associated with AD at the gene level, *CADM1*, *TUBA1B*, *CABIN1*, and *EXOC2* were associated with AD in the present study (Supplementary Table S4).

*APOE* genotype-based EWAS also showed no significant differences in the DNAm profiles between the genotype groups (Supplementary Table S5). However, three CpG sites (Chr. 6: pos. 106315395, Chr. 9: pos. 138797992, Chr. 22: pos. 43817344) from the top 10 of the EWAS between L-Control and L-AD (4) also appeared in the top 30 of the case-control EWAS for AD (Supplementary Table S4 and Figure S2). Furthermore, EWAS between L-Control and H-AD (6) identified four CpG sites on RP4-799D16.1, a lincRNA on Chr. 1, among the top 10 results (Supplementary Table S5).

## Discussion

Here, we conducted a typical case-control EWAS to compare DNAm levels between patients with AD and controls and DNAm profiles among groups stratified by *APOE* genotypes. However, AD-associated DNAm profiles did not significantly differ, suggesting that blood-derived DNAm profiles do not reflect AD pathology. Our results are consistent with previous studies findings of limited DNAm differences in the peripheral blood of patients with AD, despite documented changes in brain tissues [35]. Our post hoc finding of ~ 13.8% statistical power indicated that the absence of significant EWAS findings reflected the limited size of the sampler rather than the absence of true biological differences. Previous studies have highlighted the tissue specificity of DNAm, suggesting that peripheral measures may not reliably capture central nervous system pathology. Although recent large-scale studies have identified immune-related DNAm signatures in blood [36, 37] these findings may heavily depend on the cohort size, analytical methods, or disease stage. We applied CDMV probe-based sequencing (~ 1.5 M CpG sites), but with only ~ 110,000 overlapping DNAm microarrays (Illumina Infinium HumanMethylation450 [HM450] and HumanMethylationEPIC [EPIC]). This coverage was limited to known AD-associated sites and possibly contributed to a lack of replication. Collectively, the present and previous findings suggest that *CADM1* [38, 39], *TUBA1B* [40] and *EXOC2* [41, 42] are involved in AD pathophysiology, *via* synaptic adhesion, microtubule stability, and vesicular transport. Furthermore, the results of blood-derived DNA might not fully reflect brain-specific methylation changes.

The absence of significant CpG findings in the present study may be due to several factors. The participants were selected based on clinical diagnoses that were confirmed an average of 3.15 years ago. Given the progressive nature of AD and the uncertainty of its biological onset, disease stages might have differed among the patients. This would consequently limit the sensitivity to detect consistent DNAm changes. Peripheral blood may not adequately capture AD-related epigenetic signals, especially when neuroinflammatory or neuronal changes are localised in the brain. Moreover, patient heterogeneity in terms of medication, comorbidities, and lifestyles may contribute to the background variability in DNAm profiles [43].

Future studies should consider longitudinal sampling and more refined disease staging to elucidate the real potential of blood-based DNAm biomarkers in AD. The combination of DNAm data with transcriptomic, proteomic, or neuroimaging information may help to uncover integrated biomarker signatures. Furthermore, targeting early-stage or genetically defined AD subtypes may enhance signal detection. Incorporating measures such as epigenetic clocks or peripheral inflammatory markers in longitudinal designs could also improve sensitivity to dynamic or early-stage changes in DNAm associated with AD pathogenesis.

### Limitations

This study has some limitations, including its modest sample size and cross-sectional design. Moreover, the reliance on clinical diagnosis without pathological confirmation may lead to misclassification. Despite these limitations, our findings highlight challenges in the identification of blood-based DNAm biomarkers for AD and underscore the need for more targeted and longitudinal approaches.

## Supplementary Information

Below is the link to the electronic supplementary material.

Supplementary Material 1

Supplementary Material 2

## Data Availability

Individual-level data cannot be made publicly available due to restrictions in the informed consent. However, researchers may access the data by entering into a collaborative research agreement with the authors or by applying for data access through the Tohoku Medical Megabank Organization in the future, subject to institutional review and approval.The summarized EWAS data from this study have been deposited in the iMETHYL database (http://imethyl.iwate-megabank.org/genomebrowser.html) and are available for download at: http://imethyl.iwate-megabank.org/download_AlzheimerEWAS.html. Access to the data requires submission of a Data Transfer Agreement (DTA) form available at the same link.

## Abbreviations

AD: Alzheimer’s disease
AMED: Japan Agency for Medical Research and Development
BBJ: BioBank Japan
CDMV: Common DNA methylation variation
DNAm: DNA methylation
EWAS: Epigenome-wide association study
GWAS: Genome-wide association study
H-AD: Patients at high risk of Alzheimer’s disease
H-Control: High-risk control
L-AD: Patients at low risk of Alzheimer’s disease
L-Control: Low-risk control
MCI: Mild cognitive impairment
NK cells: Natural killer cells
PCR: Polymerase chain reaction
SD: Standard deviation
SNP: Single nucleotide polymorphism
