# Supplementary material for: Exploring novel blood-based DNA methylation biomarkers for alzheimer’s disease via targeted sequencing of highly variable CpG sites
Source: BMC Res Notes. 2025 Aug 12;18:350. doi: 10.1186/s13104-025-07417-7 (PMC12341124; doi:10.1186/s13104-025-07417-7)
Supplement: Supplementary file 2 — Supplementary Material 2 [file 13104_2025_7417_MOESM2_ESM.docx]

**Additional file 1**

- **Supplementary Methods**

**Evaluation of genomic DNA quality**

Genomic DNA purity, yield, and fragmentation were evaluated using a 2000/2000c spectrophotometer (Thermo Fisher Scientific, Waltham, MA, USA), a Qubit 2.0 Fluorometer (Thermo Fisher Scientific), and an Agilent 2200 TapeStation (Agilent Technologies, Santa Clara, CA, USA).

**PCR primer design and pyrosequencing genotyping for AD-associated SNPs**

Primers for PCR amplification were designed using the PyroMark assay design 2.0 software (QIAGEN) supplied along with the PyroMark Instrument (QIAGEN). Genotypic analysis with the PyroMark Instrument (QIAGEN) was performed according to the manufacturer’s instructions using genomic DNA diluted to 5 ng/µL. PCR amplification was conducted in a Veriti Thermal Cycler (Thermo Fisher Scientific Inc.) with a profile of 95°C for 15 min; 45 cycles of 94°C for 30 s, 60°C for 30 s, and 72 °C for 30 s. Finally, the PCR products were incubated at 72°C for 10 min before SNP analysis was conducted on a PyroMark Instrument.

Because PCR primers for *APOE* (rs429358 and rs7412) could not be successfully designed using primer design software, we used the TaqMan SNP Genotyping Assay Human (Assay ID: C_3084793_20 and C_904973_10, Thermo Fisher Scientific) for *APOE* (rs429358 and rs7412) genotyping analysis. In accordance with the manufacturer’s instructions, genotype analysis with the TaqMan assay kit was conducted using genomic DNA diluted to 1 ng/µL. TaqMan assay was performed on the StepOnePlus Real-Time PCR System (Thermo Fisher Scientific) with a profile of 60°C for 30 s; 95°C for 5 min; 40 cycles of 95°C for 5 s, and 60°C for 30 s.

**Calculation of DNAm levels and statistical analysis**

Illumina adaptor sequences and reads <20 bp were removed from the raw sequencing data using Trim Galore v0.6.5. The remaining sequences were mapped to the human reference genome GRCh37 using NovoAlign v4.02.02^1,2^ (Novocraft Technologies Sdn. Bhd., Selangor, Malaysia), and duplicate amplicons were removed using SAMtools v1.3.1^3,4^. Overlapping paired-end reads were clipped using BamUtil v1.0.13^5^. Next, DNAm levels (%DNAm) in detectable CpGs were calculated using NovoMethyl v1.07^1,2^ (Novocraft). Of the detected CpGs, those with <6× and >300× read depths were excluded from further analyses.

Logistic regression analysis was conducted using the *glm* function in R for comparison between the two groups, with group attributes as the dependent variable, DNAm levels as the explanatory variable, and the association between the first to fifth principal component scores as correction terms. CpGs that met one of the following conditions were excluded: 1) undetectable DNAm levels in more than half of the individuals, 2) high levels of deficiency within one group, and 3) CpG sites for which the standard deviation (SD) could not be calculated or was zero. Multiple testing corrections were performed using Bonferroni correction.

**Data processing before EWAS**

DNAm profile data were processed using R v4.0.5 (R Foundation for Statistical Computing, Vienna, Austria). The *estimateCellCounts* function in the R package *Minfi*^6^ was used to estimate the cell-type composition and correction in the epigenome-wide association study (EWAS). Whole-genome bisulfite sequencing data of the six blood cell types (monocytes, neutrophils, CD4^+^T cells, CD8^+^T cells, B cells, and NK cells) we have obtained from iMETHYL database were referenced to extract utilised CpG sites and estimate cell type composition from the data analysed in the present study, and "Coefficients" we recalculated. In the acquired DNAm data, CpG sites with no detectable DNAm profiles in more than half of the individuals and those with no variation in DNAm levels between individuals (standard deviation (SD) = 0) were excluded from the analysis to create a dataset for the EWAS. Next, principal component analysis was conducted using the *Prcomp* function in R. DNAm levels at each CpG site were normalised to a mean of 0 and a standard deviation of 1. Finally, the pairwise association between the first to fifth principal component scores and estimates of cell type composition were conducted using a single regression with the *lm* function to control for multicollinearity in the EWAS.

- **Supplementary Figures**

**Supplementary Figure S1.**

**Principal component analysis (PCA) of common detectable DNAm profiles.** Scatter plots of DNAm profiles in two-dimensional PCA based on *APOE* risk alleles (rs429358 and rs7412) with or without AD are shown as follows: **a.** PC1 vs PC2. **b.** PC3 vs PC4. H-AD, High-risk AD, L-AD; Low-risk AD, H-Ctrl; High-risk healthy control; L-Ctrl; Low-risk healthy control. **c.** Proportion of variance from PC1 to PC20.


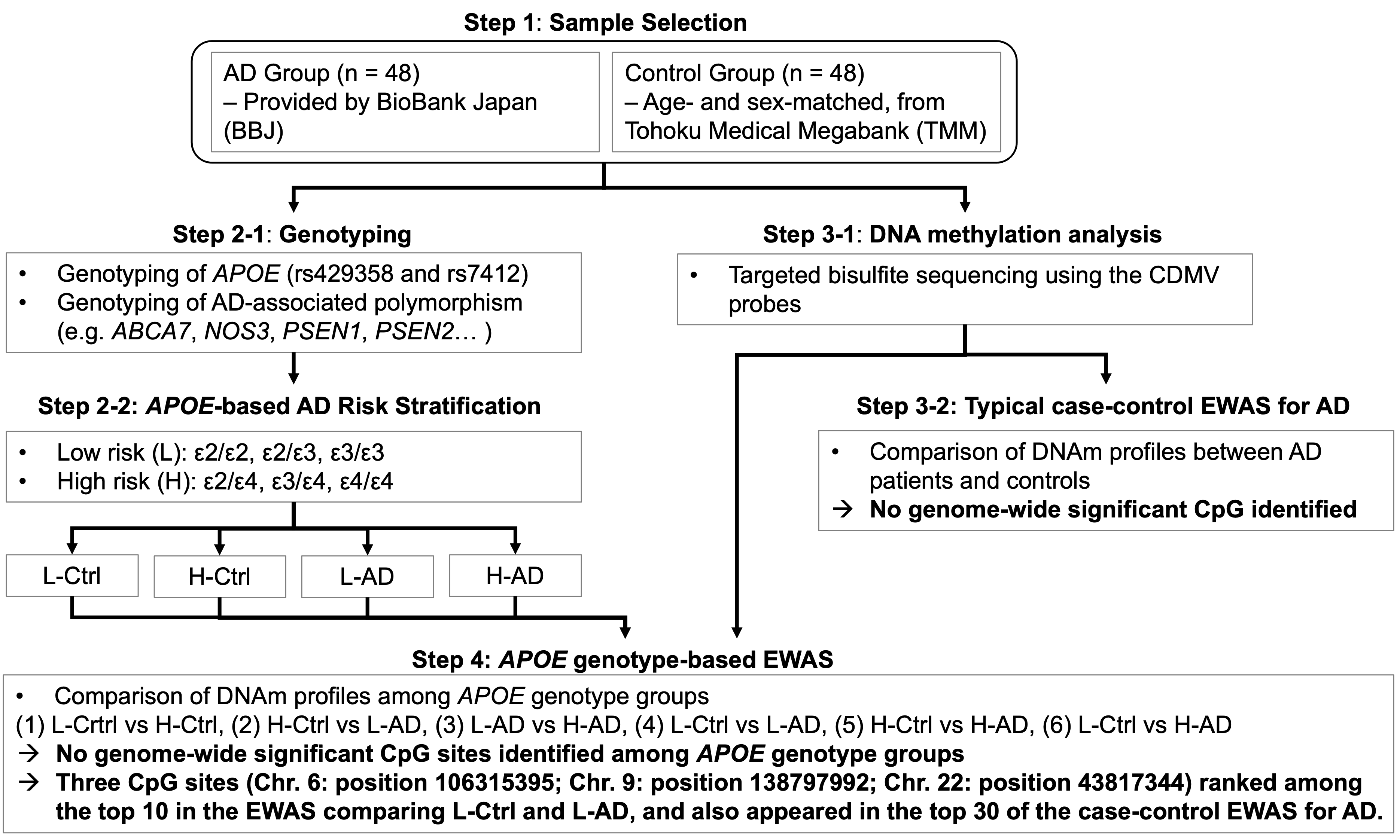


**Supplementary Figure S2.**

**Schematic summary of sample stratification and analysis steps.**

**REFERENCES**

1. Hatem, A., Bozdağ, D., Toland, A. E. & Çatalyürek, Ü. V. Benchmarking short sequence mapping tools. *BMC Bioinformatics* **14**, 184 (2013).

2. Thankaswamy-Kosalai, S., Sen, P. & Nookaew, I. Evaluation and assessment of read-mapping by multiple next-generation sequencing aligners based on genome-wide characteristics. *Genomics* **109**, 186–191 (2017).

3. Li, H. *et al.* The Sequence Alignment/Map format and SAMtools. *Bioinformatics* **25**, 2078–2079 (2009).

4. Danecek, P. *et al.* Twelve years of SAMtools and BCFtools. *Gigascience* **10**, (2021).

5. Jun, G., Wing, M. K., Abecasis, G. R. & Kang, H. M. An efficient and scalable analysis framework for variant extraction and refinement from population-scale DNA sequence data. *Genome Res* **25**, 918–925 (2015).

6. Aryee, M. J. *et al.* Minfi: A flexible and comprehensive Bioconductor package for the analysis of Infinium DNA methylation microarrays. *Bioinformatics* **30**, 1363–1369 (2014).
